# Artificial insemination of Holstein heifers with sex-sorted semen during the hot season in a subtropical region

**DOI:** 10.1007/s11250-017-1311-y

**Published:** 2017-05-20

**Authors:** Lian-Ben Chang, Chih-Jen Chou, Jia-Shian Shiu, Po-An Tu, Shi-Xuan Gao, Shao-Yu Peng, Shinn-Chih Wu

**Affiliations:** 1 Department of Animal Science and Technology, National Taiwan University, No. 50, Lane 155, Sec. 3, Keelung Road, Taipei, 10672 Taiwan Republic of China; 2 Institute of Biotechnology, National Taiwan University, No. 81, Chang-Xing St, Taipei, 10672 Taiwan Republic of China; 3 Agriculture Experiment Station, College of Bioresources and Agriculture, National Taiwan University, No. 50, Lane 155, Sec. 3, Keelung Road, Taipei, 10672 Taiwan Republic of China; 4 Hsinchu Branch, Livestock Research Institute, Council of Agriculture, Executive Yuan, No. 207-5, Bi-tou-mian, Wu-hoo Village, Si-hoo Township, Miao-li County, 36848 Taiwan Republic of China; 5 Department of Animal Science, National Pingtung University of Science and Technology, 1, Shuefu Road, Neipu, Pingtung, 91201 Taiwan Republic of China

**Keywords:** Holstein heifers, Sex-sorted semen, Insemination deposition, Pre-ovulation follicle, Subtropical regions

## Abstract

Our aim was to investigate insemination techniques in order to improve pregnancy rates of artificial insemination (AI) using sex-sorted semen (sexed AI) in cattle in tropical and subtropical (T/ST) regions. In T/ST regions, the pregnancy rates by sexed AI are reportedly the lowest in the hottest months of the year, with less than 15% in cows and 35–40% in heifers (PMID 24048822). We compared sexed AI by depositing the semen into the uterine body (UB-AI, *n* = 12) versus the unilateral uterine horn (UUH-AI, *n* = 14) of pre-ovulation heifers. The ovary and follicle were assessed by rectal ultrasound before AI. After insemination, pregnancy was determined by ultrasound at approximately 40 days and approximately 70 days. In the present study, we demonstrated that high pregnancy rates (>70%) by sexed AI in the hottest season in a subtropical region such as Taiwan can be achieved when heifers with pre-ovulation follicles are used. The overall pregnancy rates were 54% higher in the UUH-AI (71%) group than in the UB-AI (42%) group (*P* = 0.06), examined on approximately 40 days post-sexed AI. Surprisingly, however, the pregnancy outcome appeared to be higher in the hot season (62%) than in the cool season (46%) although this difference was not statistically significant. Based on the present study, we recommend that cattle breeders perform UUH-AI using sex-sorted semen for heifers with pre-ovulation follicles in order to achieve satisfactory pregnancy outcome in the hot seasons in T/ST regions.

## Introduction

Sex-sorted semen is now widely used in cattle breeding to enhance industrial competitiveness (Rath and Johnson 2008) and achieve high female fertility (Seidel 2003a, b). X chromosome sex-sorted semen with artificial insemination (AI) can effectively control the sex ratio of the offspring of dairy cows. In the process of flow cytometry sorting, a number of insults including sperm dilution, centrifugation, dyeing, pressure, laser, discharge, and other processes often lead to damage to sperm structure, decreasing its viability. Consequently, AI using sex-sorted semen (sexed AI) has a pregnancy rate lower than that using conventional unsorted semen (Carvalho et al. 2010; DeJarnette et al. 2011). In addition, the process of flow cytometry sorting is very slow, at a rate of 10–18 million/h. A very low number (~2.0 million) of sperm is packaged in a sex-sorted semen straw only 10% that in conventional unsorted semen (Seidel 2011). The pregnancy rate of sexed AI is approximately 70–75% of that of sex-unsorted semen (DeJarnette et al. 2009). Despite the relatively low pregnancy outcome, the dairy industry is witnessing a trend of switching to sexed AI; this is in part due to higher than 90% female rates in calves (Healy et al. 2013).

Previous studies indicate that high temperature has a negative effect on the reproduction process of dairy cows owing to undetected estrous cycle, decreased estrous period intensity (De Rensis and Scaramuzzi 2003; De Rensis et al. 2015), effects on the physical activity (García-Ispierto et al. 2006; García-Ispierto et al. 2007), and low fertility (López-Gatius 2012; López-Gatius 2013). High environmental temperature has a detrimental effect on embryo survival (García-Ispierto et al. 2006) as well as on the survival and motility of sex-sorted sperm (García-Ispierto et al. 2007; Mellado et al. 2013). Cerchiaro et al. (2007) reported that the pregnancy rate of sexed AI in heifers was lowest in summer (44.2%) and significantly lower than that in spring (53.9%), autumn (50.8%), and winter (50.7%). Similarly, Donovan et al. (2003) reported that the pregnancy rate of sexed AI in heifers in summer (33%) was only approximately half of that in winter (64%). The pregnancy rates in dairy cows are generally lower than those in heifers, but show the same trend in that summer is the worst season: 16% in summer and 21% in winter in a recent study (Mellado et al. 2014).

Taiwan is located in a subtropical region. In 2007–2016, the average monthly temperature and relative humidity in summer reached 27.7 °C and 72.8%, respectively. Such an environment with continuous high temperatures (Table 1) and high humidity has posed a serious challenge to the reproductive performance of dairy cattle. The low amount of sex-sorted semen per straw and the compromised sperm quality conditions present a further burden. Previous studies have shown that the deposition sites and estrus status can greatly affect the outcome of sexed AI. Kurykin et al. (2007) reported that pregnancy rates are higher than 100% when the animals are in strong estrus (45.9%) than when they are in weak estrus (20.8%).

**Table 1:** Comparison pregnancy per AI (P/AI) of females inseminated with sex-sorted semen in hot regions

Area	Climate	Animal category	Pregnancy per AI based on sex-sorted semen used in AI % (*n*/*n*)	References
Florida	Subtropical region	Heifers	Winter: 64	Donovan et al. 2003
			Summer: 33	
Northern Italy	Subtropical region	Heifers	Spring: 53.9 (82/152)	Cerchiaro et al. 2007
			Summer: 44.2 (19/43)	
			Autumn: 50.8 (62/122)	
			Winter: 50.7 (111/219)	
Northern Mexico	Desert climate	Heifer cows	Overall: 41.6	Mellado et al. 2014
			Winter: 21	
			Summer: 16	
			Overall: 17.3	

To date, few studies have sought to improve sexed AI in dairy cattle in the subtropical regions. The aim of the present study was to assess the effects of animal status, deposition site, and season of sexed AI in Taiwan. We hypothesized that heifers with obvious estrous cycle condition should have pre-ovulation follicles; hence, by monitoring pre-ovulation follicles, the pregnancy rate using sex-sorted semen can be improved. We further compared the effects of different deposition sites of pregnancy rates.

## Materials and methods

### Handling and management of experimental animals

We selected 26 heifers with pre-ovulation follicles after spontaneous estrus for 8–12 h. Heifers 12 to 14 months old with body weight between 355 and 410 kg and normal estrous cycle were also chosen. They were housed at the ranch of the National Taiwan University (Agriculture Experiment Station, College of Bioresources and Agriculture). During the experiment, heifers were fed twice a day (05:00 and 16:00), and estrus was observed four times a day (05:00, 11:00, 16:00, and 22:00) for 20 to 30 min daily. Heifers with spontaneous estrus after 8–12 h were inseminated with sex-sorted semen. We used sex-sorted semen from a single sire (Frontier Agriculture Systems, Inc., Taipei, Taiwan). There were approximately 2.3 million sperm per straw of sex-sorted semen. AI of heifers was performed by a professional. Before insemination, the ovary and follicle were assessed by rectal palpation and rectal ultrasound (portable ultrasound scanner equipped with a 5.0-MHz linear array transducer; SonoSite, Inc., USA). After diagnosis, we were able to confirm deposition in the pre-ovulatory follicle. After thawing the semen for approximately 30 s at 36 °C, a small amount of semen was examined to confirm if it was still viable for AI. Twenty-six heifers were randomly assigned into two groups: (1) sexed AI to uterus body pre-ovulation (UB-AI, *n* = 12) and (2) sexed AI to unilateral uterine horn pre-ovulation (UUH-AI, *n* = 14). Pregnancy was determined by ultrasound at approximately 40 days and approximately 70 days after insemination. We recorded the pregnancy and embryonic loss rates during the pregnancy period and the live birth of calves and female rates after parturition.

### Statistical analysis

SAS (Statistical Analysis Systems Institute Inc., Cary, NC, USA) was used in the analysis. Chi-squared analysis (*χ* ^2^) was used for comparing the results of different depositions of semen and insemination in the cool season or the hot season in relation to pregnancy rate. Differences were considered statistically significant when *P* <0.05.

## Results

### Diameter of pre-ovulation follicles

**Fig. 1:**
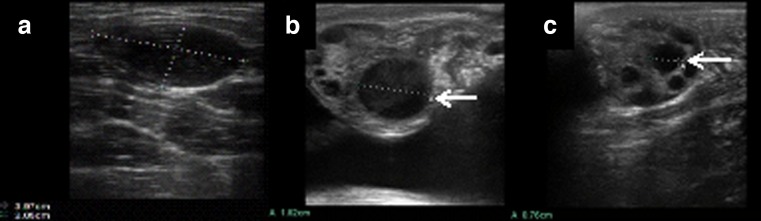
There is no follicle in the ovary via ultrasound detection (**a**), there is a dominant follicle (18.2 mm, *arrow*) in the ovary via ultrasound detection (**b**), and there is a small follicle (7.6 mm, *arrow*) in the ovary via ultrasound detection (**c**)

An ultrasound scan can be used to observe the structure of spontaneous ovarian cycle, with the follicle including the corpus luteum and ovarian stroma. Ovarian stroma imaged via ultrasound showed no follicles (Fig. 1a). Dominant follicles measure 18.2 mm in diameter (Fig. 1b). A small follicle (7.6 mm) in the ovary is also detected via ultrasound (Fig. 1c). The pre-ovulation follicles of all heifers were between 12 and 18.2 mm in our experiment. Follicle diameter of heifers in the cool and hot seasons is shown in Table 2. The average diameter of pre-ovulation follicles was 13.8 mm.

**Table 2:** Follicle diameter of heifers in cool and hot season

Follicle diameter (mm)	Follicle diameter of all heifers	Follicle diameter of pregnant heifers	Follicle diameter of not pregnant heifers
In hot season	13.3 ± 1.4	13.1 ± 1.5	13.6 ± 1.5
In cool season	14.2 ± 1.9	14.5 ± 2.3	14 ± 1.5

### Pregnancy rate, embryonic loss rate, live-born calf rate, and live-born heifer rate

**Fig. 2:**
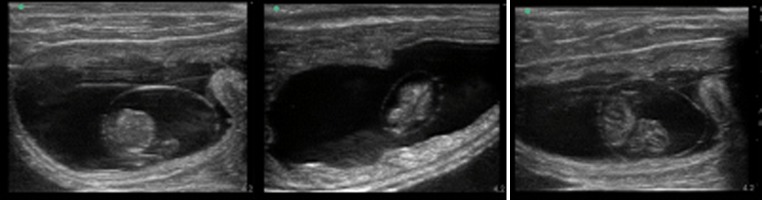
Pregnancy of heifer on approximately 40 days after inseminated with sex-sorted semen

**Fig. 3:**
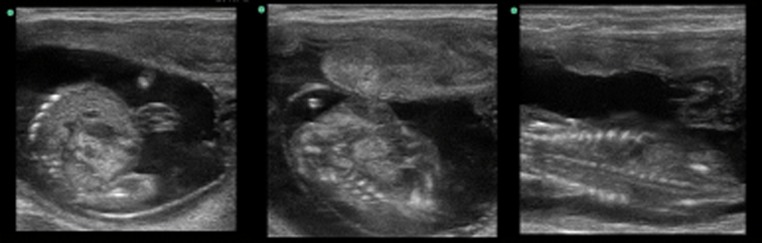
Pregnancy of heifer on approximately 70 days after inseminated with sex-sorted semen

Sex-sorted semen was inseminated in the UB-AI and UUH-AI groups after approximately 40 and 70 days. The pregnancy and embryonic loss rates are shown in Table 3. The pregnancy rates of sex-sorted semen after heifer insemination at approximately 40 days in the UB-AI and UUH-AI groups were 42 and 71%, respectively (*P* = 0.06) (Fig. 2). The pregnancy rates after heifer insemination at approximately 70 days of the UB-AI and UUH-AI groups were 42 and 64%, respectively (*P* = 0.32) (Fig. 3). The embryonic loss rate was 7% in our experiment. The number of live-born calves and live-born heifers inseminated with sex-sorted semen are also shown in Table 3. The female ratio of live calves after confirmation was 93% in our experiment.

**Table 3:** Pregnancy rate、embryonic loss rate、live offspring rate, and female rate of heifers on approximately 40 and 70 days after frozen-thawed sex-sorted semen deposition at UB and UUH with pre-ovulatory follicle

Site of deposition^a^	UB-AI, *n* (%)	UUH-AI, *n* (%)	In total, *n* (%)
Number of AI	12	14	26
Number of pregnant heifers at approximately 40 days (% per AI)	5 (42)	10 (71)	15 (58)
Number of pregnant heifers at approximately 70 days (% per AI)	5 (42)	9 (64)	14 (54)
Number of embryonic loss rate (% per AI)	0 (0)	1 (10)	1 (7)
Number of live offspring rate (% per born)	5 (100)	9 (100)	14 (100)
Number of female (% offspring rate)	5 (100)	8 (89)	13 (93)

*UB* semen deposition in the uterine body, *UUH* semen deposition in the unilateral uterine horn

^a^Deposition sites within the uterus

### Pregnancy rates of heifers inseminated in the cool season and the hot season

Temperature and humidity index during the cool and the hot seasons in Taipei, Taiwan from 2007 to 2016 were measured. The average temperature and humidity index in the hot season were 27.7 ± 2.3 °C and 72.8 ± 3.5%, respectively. The maximum temperature and humidity index were 36.0 ± 1.9 °C and 95.0 ± 3.5%, respectively. Sex-sorted semen was UB-AI and UUH-AI in the cool season and the hot season. The resulting pregnancy rates were compared. The pregnancy rates of heifers inseminated in the cool season and the hot season were 46 and 62%, respectively (*P* = 0.43). There was no significant effect in the interaction between different sites of semen deposition in the cool season and hot season and pregnancy rate (*P* = 0.58).

## Discussion

In the early study, the three critical parameters for measurement of follicular growth by trans-rectal ultrasound are follicle appearance (4 mm), follicle growth (9 mm), and pre-ovulatory follicles (from 10 to 20 mm) (Lucy 2007). In our experiment, if the follicle diameter was less than 10 mm via ultrasound image detection, the AI procedure was not implemented. Sex-sorted semen was used to inseminate heifers with pre-ovulation follicles after spontaneous estrus for 8–12 h. Seidel and Schenk (2008) reported that 1 million sperm per straw of sex-sorted semen was inseminated in the UB-AI and UUH-AI groups by estrus synchronization. The pregnancy rates of heifers after 28 to 31 days and 56 to 58 days in the UB-AI and UUH-AI groups were 46, 32, 39, and 28%, respectively. In their study, 3 million sperm per straw of sex-sorted semen was in the UB-AI and UUH-AI groups by additional estrus synchronization. After 28 to 31 days and 56 to 58 days, the pregnancy rates of heifers in the UB-AI and UUH-AI groups were 47, 51, 44, and 43%, respectively. Using different amounts of sex-sorted sperm in their experiment, it was determined whether 1 million sperm per straw in the UUH-AI had significant declined pregnancy rate than other groups after inseminated 56 to 58 days.

Bodmer et al. (2005) found that 2 million sex-sorted sperm are inseminated in the UB of heifers and cows by non-estrus synchronization. The pregnancy rates of heifer insemination after 30 to 40 days and 70 to 90 days were 33.3 and 29.6%, respectively, whereas the pregnancy rates of cows were 27.6 and 23.8%. In their experiment, the pregnancy rate following heifer insemination with sex-sorted semen was slightly higher than that for cows, but there was no significant difference. Further, in their experiment, the pregnancy rates following heifer insemination after 30 to 40 days and 70 to 90 days were slightly lower than our 2.3 million sperm per straw of sexed AI in the UB-AI group (42 and 42%). A possible reason is that the amount of sperm in their experiment was 0.3 million sperm per straw lower than that in our experiment. Therefore, one of the main factors influencing a higher pregnancy rate is a higher sperm count in a single straw (Seidel and Schenk 2008).

In a study by Kurykin et al. (2007), estrus synchronization in heifer insemination was conducted using approximately 2.2 million sperm per straw of sex-sorted semen in the UB, UUH, and utero-tubal junction. In their experiment, heifers exhibiting strong signs of estrus reached 45.9%, but weak signs of estrus only reached 20.8% of pregnancy rate. Thus, strong or weak signs of estrus affect pregnancy rate. In most heifers with weak signs of estrus, it is not possible to evaluate whether ovulation time is appropriate for AI; thus, when using fewer sex-sorted sperm, the key lies in the timing of fertilization (Kurykin et al. 2003).

In Taiwan, the hot season mostly is with high temperature and high humidity. The pregnancy rate had no significant effect on heifers inseminated with sex-sorted semen in the cool and hot seasons. In the annual research of Mellado et al. (2014), the pregnancy rate of heifers inseminated using sex-sorted semen was significantly higher than that of cows; there was no significant effect of insemination using sex-sorted semen on the pregnancy rate of heifers in the cool season and the hot season. This result is similar to that of our experiment; there was no significant effect of insemination using sex-sorted semen on the pregnancy rate of heifers in the cool season or the hot season in subtropical regions. The possible reason was that the heifers had obvious signs of estrus and had pre-ovulation follicles under hot stress. We used sexed AI in heifers with pre-ovulation follicles after spontaneous estrus for 8–12 h, and no reduction in pregnancy rate was observed in the hot season. The possible reason was that the heifers were not under lactation pressure or uterine without involution.

Most studies did not mention rates of live-born calves using sexed AI. There were equally live calves born using AI on lactating cows by sex-unsorted semen using the estrus synchronized in the research of Stewart et al. (2011). It was clear that AI using sex-sorted semen did not affect the performance of live-born calves. The proportion of live female calves after confirmation was 93% in this experiment. This result was similar to the proportion of females (93.2%) following the use of sex-sorted semen in the estrus synchronization program in the experiment of Kurykin et al. (2007). Other studies showed that the proportion of females following the use of sexed AI in lactating cows can reach 90% (DeJarnette et al. 2009; Norman et al. 2010).

In conclusion, heifer breeders should perform UUH-AI using sex-sorted semen on heifers that have pre-ovulation follicles to achieve satisfactory pregnancy outcomes in subtropical regions. Large-scale follow-up studies are needed to confirm these findings.
